# Supplementary material for: Antimicrobial resistance and genome characteristics of Salmonella enteritidis from Huzhou, China
Source: PLoS One. 2024 Jun 4;19(6):e0304621. doi: 10.1371/journal.pone.0304621 (PMC11149840; doi:10.1371/journal.pone.0304621)
Supplement: S1 Table — (DOCX) [file pone.0304621.s001.docx]

The information of 43 *Salmonella enteritidis* isolates strain

| Strain number | Region | specimen origin | Date of separation | Serotype |
| --- | --- | --- | --- | --- |
| S2023121 | Changxing | patient stool | 2023.6.10 | *S.enteritidis* |
| S2023204 | Wuxing | patient stool | 2023.7.8 | *S.enteritidis* |
| S2023229 | Anji | patient stool | 2023.7.16 | *S.enteritidis* |
| S2023230 | Anji | patient stool | 2023.7.20 | *S.enteritidis* |
| S2023231 | Anji | patient stool | 2023.8.13 | *S.enteritidis* |
| S2023232 | Anji | patient stool | 2023.8.13 | *S.enteritidis* |
| S2023233 | Anji | patient stool | 2023.8.26 | *S.enteritidis* |
| S2023420 | Wuxing | patient stool | 2023.9.3 | *S.enteritidis* |
| S2023200 | Wuxing | patient stool | 2023.7.3 | *S.enteritidis* |
| S2021064 | Deqing | patient stool | 2021.3.31 | *S.enteritidis* |
| S2021145 | Wuxing | patient stool | 2021.5.11 | *S.enteritidis* |
| S2021154 | Anji | patient stool | 2021.5.17 | *S.enteritidis* |
| S2021237 | Wuxing | patient stool | 2021.6.17 | *S.enteritidis* |
| S2021245 | Wuxing | patient stool | 2021.6.23 | *S.enteritidis* |
| S2021270 | Wuxing | patient stool | 2021.6.29 | *S.enteritidis* |
| S2021272 | Wuxing | patient stool | 2021.6.30 | *S.enteritidis* |
| S2021332 | Wuxing | patient stool | 2021.7.15 | *S.enteritidis* |
| S2021344 | Changxing | patient stool | 2021.7.20 | *S.enteritidis* |
| S2022111 | Deqing | patient stool | 2022.5.12 | *S.enteritidis* |
| S2022121 | Wuxing | patient stool | 2022.5.23 | *S.enteritidis* |
| S2022122 | Wuxing | patient stool | 2022.5.24 | *S.enteritidis* |
| S2022123 | Wuxing | patient stool | 2022.5.24 | *S.enteritidis* |
| S2022601 | Changxing | patient stool | 2022.9.26 | *S.enteritidis* |
| S2022602 | Changxing | patient stool | 2022.9.26 | *S.enteritidis* |
| S2022603 | Changxing | patient stool | 2022.9.26 | *S.enteritidis* |
| S2022604 | Changxing | patient stool | 2022.9.26 | *S.enteritidis* |
| S2022605 | Changxing | patient stool | 2022.9.26 | *S.enteritidis* |
| S2022606 | Changxing | patient stool | 2022.9.26 | *S.enteritidis* |
| S2022607 | Changxing | patient stool | 2022.9.26 | *S.enteritidis* |
| S2022608 | Changxing | patient stool | 2022.9.26 | *S.enteritidis* |
| S2022609 | Changxing | patient stool | 2022.9.26 | *S.enteritidis* |
| S2022610 | Changxing | patient stool | 2022.9.26 | *S.enteritidis* |
| S2022643 | Wuxing | patient stool | 2022.9.28 | *S.enteritidis* |
| S2022645 | Anji | patient stool | 2022.10.11 | *S.enteritidis* |
| S2022790 | Nanxun | patient stool | 2022.10.29 | *S.enteritidis* |
| S2022791 | Nanxun | patient stool | 2022.10.31 | *S.enteritidis* |
| S2023066 | Anji | patient stool | 2023.3.20 | *S.enteritidis* |
| S2023144 | Wuxing | patient stool | 2023.5.18 | *S.enteritidis* |
| S2023209 | Changxing | patient stool | 2023.5.31 | *S.enteritidis* |
| S2023561 | Wuxing | patient stool | 2023.8.17 | *S.enteritidis* |
| S2023828 | Deqing | patient stool | 2023.9.28 | *S.enteritidis* |
| S20231004 | Anji | food | 2023.11.16 | *S.enteritidis* |
| S20231006 | Anji | food | 2023.11.16 | *S.enteritidis* |
