# Supplementary material for: Antimicrobial resistance and genome characteristics of Salmonella enteritidis from Huzhou, China
Source: PLoS One. 2024 Jun 4;19(6):e0304621. doi: 10.1371/journal.pone.0304621 (PMC11149840; doi:10.1371/journal.pone.0304621)
Supplement: S2 Table — (DOCX) [file pone.0304621.s002.docx]

Genome assembly profile of *Salmonella enteritidis*

| Assembly | Total length | GC (%) |
| --- | --- | --- |
| S2021064 | 4465346 | 52.42 |
| S2021145 | 4447915 | 52.42 |
| S2021154 | 4418330 | 52.41 |
| S2021237 | 4447741 | 52.42 |
| S2021245 | 4451920 | 52.42 |
| S2021270 | 4451825 | 52.42 |
| S2021272 | 4417739 | 52.42 |
| S2021332 | 4419819 | 52.42 |
| S2021344 | 4459788 | 52.41 |
| S2022111 | 4722932 | 52.1 |
| S2022121 | 4728913 | 52.14 |
| S2022122 | 4716985 | 52.1 |
| S2022123 | 4720767 | 52.1 |
| S2022601 | 4691505 | 52.14 |
| S2022602 | 4693620 | 52.14 |
| S2022603 | 4693213 | 52.14 |
| S2022604 | 4691056 | 52.13 |
| S2022605 | 4691312 | 52.13 |
| S2022606 | 4691905 | 52.14 |
| S2022607 | 4691716 | 52.14 |
| S2022608 | 4691809 | 52.14 |
| S2022609 | 4691169 | 52.13 |
| S2022610 | 4691080 | 52.13 |
| S2022643 | 4726039 | 52.1 |
| S2022645 | 4757703 | 52.09 |
| S2022790 | 4757525 | 52.09 |
| S2022791 | 4755011 | 52.08 |
| S2023066 | 4693157 | 52.14 |
| S20231004 | 4750622 | 52.08 |
| S20231006 | 4750737 | 52.08 |
| S2023121 | 4722971 | 52.11 |
| S2023144 | 4715212 | 52.11 |
| S2023200 | 4729101 | 52.09 |
| S2023204 | 4723814 | 52.1 |
| S2023209 | 4739690 | 52.07 |
| S2023229 | 4719720 | 52.11 |
| S2023230 | 4719648 | 52.11 |
| S2023231 | 4721042 | 52.12 |
| S2023232 | 4721011 | 52.12 |
| S2023233 | 4727189 | 52.1 |
| S2023420 | 4724578 | 52.1 |
| S2023561 | 4733371 | 52.15 |
| S2023828 | 4725929 | 52.11 |
