# Supplementary material for: Antimicrobial resistance and genome characteristics of Salmonella enteritidis from Huzhou, China
Source: PLoS One. 2024 Jun 4;19(6):e0304621. doi: 10.1371/journal.pone.0304621 (PMC11149840; doi:10.1371/journal.pone.0304621)
Supplement: S3 Table — (DOCX) [file pone.0304621.s003.docx]

C Results of MLST typing of *Salmonella enteritidis* genome

| ID | ST | aroC | dnaN | hemD | hisD | purE | sucA | thrA |
| --- | --- | --- | --- | --- | --- | --- | --- | --- |
| S2021064 | 11 | 5 | 2 | 3 | 7 | 6 | 6 | 11 |
| S2021145 | 11 | 5 | 2 | 3 | 7 | 6 | 6 | 11 |
| S2021154 | 11 | 5 | 2 | 3 | 7 | 6 | 6 | 11 |
| S2021237 | 11 | 5 | 2 | 3 | 7 | 6 | 6 | 11 |
| S2021245 | 11 | 5 | 2 | 3 | 7 | 6 | 6 | 11 |
| S2021270 | 11 | 5 | 2 | 3 | 7 | 6 | 6 | 11 |
| S2021272 | 11 | 5 | 2 | 3 | 7 | 6 | 6 | 11 |
| S2021332 | 11 | 5 | 2 | 3 | 7 | 6 | 6 | 11 |
| S2021344 | 11 | 5 | 2 | 3 | 7 | 6 | 6 | 11 |
| S2022111 | 11 | 5 | 2 | 3 | 7 | 6 | 6 | 11 |
| S2022121 | 11 | 5 | 2 | 3 | 7 | 6 | 6 | 11 |
| S2022122 | 11 | 5 | 2 | 3 | 7 | 6 | 6 | 11 |
| S2022123 | 11 | 5 | 2 | 3 | 7 | 6 | 6 | 11 |
| S2022601 | 11 | 5 | 2 | 3 | 7 | 6 | 6 | 11 |
| S2022602 | 11 | 5 | 2 | 3 | 7 | 6 | 6 | 11 |
| S2022603 | 11 | 5 | 2 | 3 | 7 | 6 | 6 | 11 |
| S2022604 | 11 | 5 | 2 | 3 | 7 | 6 | 6 | 11 |
| S2022605 | 11 | 5 | 2 | 3 | 7 | 6 | 6 | 11 |
| S2022606 | 11 | 5 | 2 | 3 | 7 | 6 | 6 | 11 |
| S2022607 | 11 | 5 | 2 | 3 | 7 | 6 | 6 | 11 |
| S2022608 | 11 | 5 | 2 | 3 | 7 | 6 | 6 | 11 |
| S2022609 | 11 | 5 | 2 | 3 | 7 | 6 | 6 | 11 |
| S2022610 | 11 | 5 | 2 | 3 | 7 | 6 | 6 | 11 |
| S2022643 | 11 | 5 | 2 | 3 | 7 | 6 | 6 | 11 |
| S2022645 | 11 | 5 | 2 | 3 | 7 | 6 | 6 | 11 |
| S2022790 | 11 | 5 | 2 | 3 | 7 | 6 | 6 | 11 |
| S2022791 | 11 | 5 | 2 | 3 | 7 | 6 | 6 | 11 |
| S2023066 | 11 | 5 | 2 | 3 | 7 | 6 | 6 | 11 |
| S20231004 | 11 | 5 | 2 | 3 | 7 | 6 | 6 | 11 |
| S20231006 | 11 | 5 | 2 | 3 | 7 | 6 | 6 | 11 |
| S2023121 | 11 | 5 | 2 | 3 | 7 | 6 | 6 | 11 |
| S2023144 | 11 | 5 | 2 | 3 | 7 | 6 | 6 | 11 |
| S2023200 | 11 | 5 | 2 | 3 | 7 | 6 | 6 | 11 |
| S2023204 | 11 | 5 | 2 | 3 | 7 | 6 | 6 | 11 |
| S2023209 | 11 | 5 | 2 | 3 | 7 | 6 | 6 | 11 |
| S2023229 | 11 | 5 | 2 | 3 | 7 | 6 | 6 | 11 |
| S2023230 | 11 | 5 | 2 | 3 | 7 | 6 | 6 | 11 |
| S2023231 | 11 | 5 | 2 | 3 | 7 | 6 | 6 | 11 |
| S2023232 | 11 | 5 | 2 | 3 | 7 | 6 | 6 | 11 |
| S2023233 | 11 | 5 | 2 | 3 | 7 | 6 | 6 | 11 |
| S2023420 | 11 | 5 | 2 | 3 | 7 | 6 | 6 | 11 |
| S2023561 | 11 | 5 | 2 | 3 | 7 | 6 | 6 | 11 |
| S2023828 | 11 | 5 | 2 | 3 | 7 | 6 | 6 | 11 |
