# Supplementary material for: Antimicrobial resistance and genome characteristics of Salmonella enteritidis from Huzhou, China
Source: PLoS One. 2024 Jun 4;19(6):e0304621. doi: 10.1371/journal.pone.0304621 (PMC11149840; doi:10.1371/journal.pone.0304621)
Supplement: S4 Table — (DOCX) [file pone.0304621.s004.docx]

Genomic virulence gene results of *Salmonella enteritidis*

| GENE | GENE_COUNT |
| --- | --- |
| sopE2 | 43 |
| sopA | 43 |
| avrA | 43 |
| orgC | 43 |
| orgB | 43 |
| orgA | 43 |
| prgK | 43 |
| prgJ | 43 |
| prgI | 43 |
| prgH | 43 |
| sptP | 43 |
| sicP | 43 |
| sipA/sspA | 43 |
| sipD | 43 |
| sipC/sspC | 43 |
| sipB/sspB | 43 |
| sicA | 43 |
| spaS | 43 |
| spaR | 43 |
| spaQ | 43 |
| spaP | 43 |
| spaO | 43 |
| invJ | 43 |
| invI | 43 |
| invC | 43 |
| invB | 43 |
| invA | 43 |
| invE | 43 |
| invG | 43 |
| invF | 43 |
| invH | 43 |
| sifB | 43 |
| steA | 43 |
| sopD2 | 43 |
| csgG | 43 |
| csgF | 43 |
| csgE | 43 |
| csgD | 43 |
| csgB | 43 |
| csgA | 43 |
| csgC | 43 |
| sifA | 43 |
| misL | 43 |
| fimI | 43 |
| fimC | 43 |
| fimD | 43 |
| fimH | 43 |
| fimF | 43 |
| mgtC | 43 |
| sopB/sigD | 43 |
| pipB | 43 |
| ompA | 43 |
| mig-14 | 43 |
| ssaU | 43 |
| ssaT | 43 |
| ssaS | 43 |
| ssaR | 43 |
| ssaQ | 43 |
| ssaP | 43 |
| ssaO | 43 |
| ssaN | 43 |
| ssaV | 43 |
| ssaM | 43 |
| ssaL | 43 |
| ssaK | 43 |
| ssaJ | 43 |
| ssaI | 43 |
| ssaH | 43 |
| ssaG | 43 |
| sseG | 43 |
| sseF | 43 |
| sscB | 43 |
| sseE | 43 |
| sseD | 43 |
| sseC | 43 |
| sscA | 43 |
| sseB | 43 |
| sseA | 43 |
| ssaE | 43 |
| ssaD | 43 |
| ssaC | 43 |
| spiC/ssaB | 43 |
| sseL | 43 |
| sseK1 | 43 |
| lpfA | 43 |
| lpfB | 43 |
| lpfC | 43 |
| lpfD | 43 |
| sopD | 43 |
| slrP | 43 |
| ratB | 43 |
| sodCI | 43 |
| steB | 43 |
| sseJ | 43 |
| steC | 43 |
| pipB2 | 43 |
| mgtB | 42 |
| lpfE | 42 |
| sseI/srfH | 34 |
| spvR | 34 |
| spvB | 34 |
| spvC | 34 |
| pefB | 34 |
| pefC | 34 |
| pefD | 34 |
| sspH2 | 33 |
| rck | 33 |
| shdA | 24 |
