# Supplementary material for: Antimicrobial resistance and genome characteristics of Salmonella enteritidis from Huzhou, China
Source: PLoS One. 2024 Jun 4;19(6):e0304621. doi: 10.1371/journal.pone.0304621 (PMC11149840; doi:10.1371/journal.pone.0304621)
Supplement: S5 Table — (DOCX) [file pone.0304621.s005.docx]

E  *Salmonella enteritidis* genome virulence gene pathogenic system

| PRODUCT | PRODUCT_COUNT |
| --- | --- |
| type III secretion system | 3059 |
| curli | 215 |
| chaperone | 215 |
| long polar fimbrial | 214 |
| plasmid-encoded fimbriae | 102 |
| chaparone | 86 |
| type I fimbriae | 86 |
| Mg2+ transport protein | 85 |
| DNA-binding transcriptional regulator CsgD | 43 |
| minor curlin subunit precursor curli nucleator protein CsgB | 43 |
| putative autotransporter | 43 |
| fimbrial protein internal segment | 43 |
| usher protein FimD | 43 |
| outer membrane protein A | 43 |
| antimicrobial peptide resistance protein Mig-14 | 43 |
| putative outer membrane protein | 43 |
| Gifsy-2 prophage: superoxide dismutase precursor (Cu-Zn) | 43 |
| Salmonella plasmid virulence | 34 |
| resistance to complement killing | 33 |
| AIDA autotransporter-like protein | 24 |
