# Supplementary material for: Antimicrobial resistance and genome characteristics of Salmonella enteritidis from Huzhou, China
Source: PLoS One. 2024 Jun 4;19(6):e0304621. doi: 10.1371/journal.pone.0304621 (PMC11149840; doi:10.1371/journal.pone.0304621)
Supplement: Attachment — Submitted filename: PONE-D-24-09010.pdf [file pone.0304621.s006.pdf]

PLOS ONE

Antimicrobial resistance and genome characteristics of Salmonella enteritidis from Huzhou,China

--Manuscript Draft--

**Manuscript Number:**	PONE-D-24-09010
**Article Type:**	Research Article
**Full Title:**	Antimicrobial resistance and genome characteristics of Salmonella enteritidis from Huzhou,China
**Short Title:**	Antimicrobial resistance and genome characteristics of S.enteritidis
**Corresponding Author:**	Deshun Xu<br>Huzhou Centers for Disease Control and Prevention<br>Huzhou city, CHINA
**Keywords:**	Salmonella enteritidis; Antimicrobial Resistance; Whole-genome; equencing (WGS); Multilocus sequence typing (MLST)
**Abstract:**	Salmonella enteritidis is one of the main pathogens causing gastroenteritis, which causes outbreaks and sporadic. S. enteritidis infections represent an important public health problem. S. enteritidis strains isolated from diarrhea cases and food in Huzhou from February 1st 2021 to December 30th 2023, were subjected to virulence gene, drug resistance gene, genetic correlation, antibiotic resistance, and multilocus sequence typing (MLST) analyses. All the 43 S. enteritidis isolates were identified as ST11, and contain 108 virulence-related genes. Drug sensitivity analysis showed that the resistance rate of the 43 isolates to NAL was 100%, AMP and AMS was 90.70%, respectively, and the situation of multidrug resistance is serious, 81.40% strains were resistant to three or more antibacterial drugs. Genome sequencing indicated that S. enteritidis involved 23 drug resistance genes, of which 14 genes were common to 43 strains of S. enteritidis. Phylogenetic analysis based on core-genome single-nucleotide polymorphisms indicated that 43 S. enteritidis strains were divided into 3 clusters, the 10 samples from the outbreak formed an independent branch and located in cluster 3.
**Order of Authors:**	Wei Yan
	Deshun Xu
	Liping Chen
	Xiaofang Wu
**Additional Information:**	
**Question**	**Response**
**Financial Disclosure**<br><br>Enter a financial disclosure statement that describes the sources of funding for the work included in this submission. Review the submission guidelines for detailed requirements. View published research articles from *PLOS ONE* for specific examples.<br><br>This statement is required for submission and **will appear in the published article** if the submission is accepted. Please make sure it is accurate.	The author(s) received no specific funding for this work.

Powered by Editorial Manager® and ProduXion Manager® from Aries Systems Corporation

**Funded studies**<br>Enter a statement with the following details:<br>• Initials of the authors who received each award<br>• Grant numbers awarded to each author<br>• The full name of each funder<br>• URL of each funder website<br>• Did the sponsors or funders play any role in the study design, data collection and analysis, decision to publish, or preparation of the manuscript?<br><br>Did you receive funding for this work?	
**Competing Interests**<br><br>Use the instructions below to enter a competing interest statement for this submission. On behalf of all authors, disclose any competing interests that could be perceived to bias this work—acknowledging all financial support and any other relevant financial or non-financial competing interests.<br><br>This statement is required for submission and **will appear in the published article** if the submission is accepted. Please make sure it is accurate and that any funding sources listed in your Funding Information later in the submission form are also declared in your Financial Disclosure statement.<br><br>View published research articles from *PLOS ONE* for specific examples.	The authors have declared that no competing interests exist.

Powered by Editorial Manager® and ProduXion Manager® from Aries Systems Corporation

**NO authors have competing interests**<br><br>Enter: *The authors have declared that no competing interests exist.*<br><br>**Authors with competing interests**<br><br>Enter competing interest details beginning with this statement:<br><br>*I have read the journal's policy and the authors of this manuscript have the following competing interests: [insert competing interests here]*<br><br>* typeset	
**Ethics Statement**<br><br>Enter an ethics statement for this submission. This statement is required if the study involved:<br><br>• Human participants<br>• Human specimens or tissue<br>• Vertebrate animals or cephalopods<br>• Vertebrate embryos or tissues<br>• Field research<br><br>Write "N/A" if the submission does not require an ethics statement.<br><br>General guidance is provided below. Consult the submission guidelines for detailed instructions. **Make sure that all information entered here is included in the Methods section of the manuscript.**	This study was approved by the human research ethics committee of the Huzhou Center for Disease Control and Prevention. The only human material used in this study is stool samples taken from patients for routine assessment. Oral informed consent was obtained from each participant.

Powered by Editorial Manager® and ProduXion Manager® from Aries Systems Corporation

**Format for specific study types**<br><br>**Human Subject Research (involving human participants and/or tissue)**<br>• Give the name of the institutional review board or ethics committee that approved the study<br>• Include the approval number and/or a statement indicating approval of this research<br>• Indicate the form of consent obtained (written/oral) or the reason that consent was not obtained (e.g. the data were analyzed anonymously)<br><br>**Animal Research (involving vertebrate animals, embryos or tissues)**<br>• Provide the name of the Institutional Animal Care and Use Committee (IACUC) or other relevant ethics board that reviewed the study protocol, and indicate whether they approved this research or granted a formal waiver of ethical approval<br>• Include an approval number if one was obtained<br>• If the study involved *non-human primates*, add *additional details* about animal welfare and steps taken to ameliorate suffering<br>• If anesthesia, euthanasia, or any kind of animal sacrifice is part of the study, include briefly which substances and/or methods were applied<br><br>**Field Research**<br><br>Include the following details if this study involves the collection of plant, animal, or other materials from a natural setting:<br>• Field permit number<br>• Name of the institution or relevant body that granted permission	
**Data Availability**<br><br>Authors are required to make all data underlying the findings described fully available, without restriction, and from the time of publication. PLOS allows rare exceptions to address legal and ethical concerns. See the PLOS Data Policy and FAQ for detailed information.	Yes - all data are fully available without restriction

A Data Availability Statement describing where the data can be found is required at submission. Your answers to this question constitute the Data Availability Statement and **will be published in the article**, if accepted.<br><br>**Important:** Stating 'data available on request from the author' is not sufficient. If your data are only available upon request, select 'No' for the first question and explain your exceptional situation in the text box.<br><br>Do the authors confirm that all data underlying the findings described in their manuscript are fully available without restriction?	
**Describe where the data may be found in full sentences. If you are copying our sample text, replace any instances of XXX with the appropriate details.**<br><br>• If the data are **held or will be held in a public repository**, include URLs, accession numbers or DOIs. If this information will only be available after acceptance, indicate this by ticking the box below. For example: *All XXX files are available from the XXX database (accession number(s) XXX, XXX.).*<br>• If the data are all contained **within the manuscript and/or Supporting Information files**, enter the following: *All relevant data are within the manuscript and its Supporting Information files.*<br>• If neither of these applies but you are able to provide **details of access elsewhere**, with or without limitations, please do so. For example:<br><br>*Data cannot be shared publicly because of [XXX]. Data are available from the XXX Institutional Data Access / Ethics Committee (contact via XXX) for researchers who meet the criteria for access to confidential data.*<br><br>*The data underlying the results presented in the study are available from (include the name of the third party*	All relevant data are within the manuscript and its Supporting Information files.

and contact information or URL).<br>• This text is appropriate if the data are owned by a third party and authors do not have permission to share the data.<br><br>* typeset	
Additional data availability information:	

Powered by Editorial Manager® and ProduXion Manager® from Aries Systems Corporation

Manuscript

Click here to access/download;Manuscript;genome characteristics of S.enteritidis.doc

Antimicrobial resistance and genome characteristics of *Salmonella enteritidis* from Huzhou,China

Wei Yan, Deshun Xu*, Liping Chen, Xiaofang Wu

Author affiliations:

Huzhou Center for Disease Control and Prevention, Huzhou, China, 313000

Current Address:

Huzhou Center for Disease Control and Prevention, 999 Changxing Road, Huzhou, Zhejiang 313000, China

Email address:

Wei Yan: yanw1024@163.com

Deshun Xu: xds666092@126.com

Liping Chen: lipingchen1106@hotmail.com

Xiaofang Wu: xf980718@126.com

*Corresponding Author: Deshun Xu

Email address: xds666092@126.com

Abstract

Salmonella enteritidis is one of the main pathogens causing gastroenteritis, which causes outbreaks and sporadic. *S. enteritidis* infections represent an important public health problem. *S. enteritidis* strains isolated from diarrhea cases and food in Huzhou from February 1st 2021 to December 30th 2023, were subjected to virulence gene, drug resistance gene, genetic correlation, antibiotic resistance, and multilocus sequence typing (MLST) analyses. All the 43 *S. enteritidis* isolates were identified as ST11, and contain 108 virulence-related genes. Drug sensitivity analysis showed that the resistance rate of the 43 isolates to NAL was 100%, AMP and AMS was 90.70%, respectively, and the situation of multidrug resistance is serious, 81.40% strains were resistant to three or more antibacterial drugs. Genome sequencing indicated that *S. enteritidis* involved 23 drug resistance genes, of which 14 genes were common to 43 strains of *S. enteritidis*. Phylogenetic analysis based on core-genome single-nucleotide polymorphisms indicated that 43 *S. enteritidis* strains were divided into 3 clusters, the 10 samples from the outbreak formed an independent branch and located in cluster 3.

Keywords: *Salmonella enteritidis;* Antimicrobial resistance; Whole-genome equencing (WGS); Multilocus sequence typing (MLST)

Introduction

Salmonella is a zoonotic pathogen. It is also an important pathogenic factor causing foodborne diseases [2,3] and the main cause of bacterial food poisoning [4]. Contaminated food and water source or infected individuals by *Salmonella* can become the source of infection and spread to the outside, posing a serious threat to human and animal health[1].At present, more than 2 600 serotypes or variants have been identified ［5,6］,of which about 50 bacterial types are closely related to human beings. The clinical manifestations of *Salmonella* infection are complex and diverse, which can be divided into gastroenteritis type, septicemia type (typhoid type) and local infection type. In recent years, studies [7] have shown that the incidence of typhoid fever and paratyphoid fever has decreased significantly, while some non-typhoidal *Salmonella* infections have shown an upward trend. Non-typhoidal *Salmonella* is frequently associated with diarrheal illness or self-limiting gastroenteritis in humans around the world [8,9]. In globally, non-typhoidal *salmonella* causes up to 80.3 million cases of gastrointestinal diseases and 150,000 deaths each year [10]. In China, 70%-80% of foodborne diseases are caused by non-typhoidal *Salmonella*[11]. *S. enteritidis* is the main *Salmonella* serotype, accounting for 40%-60% of laboratory-confirmed *Salmonella* infections in recent years, and has been highly valued in various regions of the world [12,13].

Antimicrobial resistance remains a serious challenge in the treatment and control of *S. enterica* infections. Multidrug-resistant strains are linked to more severe disease outcomes [14] and can be passed on along the food chain, from production to consumption [15]. With the widespread use of antibiotics, the drug resistance rate of Salmonella is also increasing, which makes the drug resistance of Salmonella become more and more serious. Multi-drug resistant bacteria and even super bacteria have emerged, which lead to the emergence of new epidemiological and clinical characteristics of the disease. Therefore, as an important foodborne pathogen, the study of drug resistance of Salmonella should also be paid enough attention[16,17].

Advances in whole-genome sequencing (WGS) technology have become an economically viable alternative to conventional typing methods for outbreak investigation and public health surveillance [18]. Comparative genomics with WGS data provides insight into the genome of pathogenic bacteria, including candidate drug compounds, potential virulence determinants, mechanisms of pathogenicity, and their evolution in pathogens. As a result, this technology has been instrumental in improving diagnostics and public health microbiology [19]. Currently, this technique is widely used to investigate the outbreak of pathogenic bacteria, such as *Bacillus cereus, Escherichia coli, Vibrio parahaemolyticus, and Salmonella species* [20–23].

In Huzhou, *S. enteritidis* has been the most frequently isolated serovar from diarrhea patients and food sources in recent years[24, 25]. In this study, the whole genomes of *S. enteritidis* isolated from diarrhea patients were sequenced to obtain preliminary information about the genotypes and distribution characteristics of drug resistance genes, virulence genes, as well as the phylogenetic and evolutionary relationships among strains isolated in Huzhou. This study provides technical support for food safety risk assessment, prevention, and control of *S. enteritidis* occurrence and development in the Huzhou area.

Materials and methods

Ethics statement

This study was approved by the human research ethics committee of the Huzhou Center for Disease Control and Prevention. The only human material used in this study is stool samples taken from patients for routine assessment. Oral informed consent was obtained from each participant.

Bacterial isolates

The study examined 41 *S. enteritidis* strains isolated obtained from diarrhea patients from six active foodborne surveillance sentinel hospitals in Huzhou, Zhejiang

from February 1st 2021 to December 30th 2023(9, 18, and 14 strains per year, respectively), and 2 *S. enteritidis* strains isolated obtained from food in November 2023. All 43 strains of *S. enteritidis* were stored at −80˚C in porcelain culture storage tubes (Qingdao Haibo, China).

Antimicrobial susceptibility tests

The antimicrobial susceptibilities of the 43 *S.enteritidis* strains were tested using the broth microdilution method and classified as sensitive, intermediate, or resistant according to the Clinical and Laboratory Standards Institute (CLSI) break points for Salmonella strains. Escherichia coli ATCC 25922 was used as a control. The results were analysed according to the CLSI breakpoints. The 17 antibacterial agents tested included the macrolide (azithromycin (AZM)), quinolone and fluoroquinolone (Nalidixic acid (NAL) and ciprofloxacin (CIP)), aminoglycoside (Amikacin (AMI) and streptomycin (STR)), Phenylpropanols (chloramphenicol (CHL)), tetracycline (tetracycline (TET) and Tigecycline (TIG)), beta-lactams (ampicillin (AMP),ampicillin/sulbactam (AMS), ceftazidime (CAZ), Ceftazidime/avibactam (CZA), cefotaxime (CTX), Ertapenem (ETP) and Meropenem (MEM)), Folate pathway antagonists (compound neomycin (SXT)) and Lipopeptides (polymyxin (CT)) classes of antibiotics.

Whole genome sequencing and assembly

The genomes of *S. enteritidis* strains were extracted using bacterial genomic DNA extraction ki. the whole genome was sequenced using a NextSeq 550 sequencer (Illumina, USA). FastQC (v0.11.9) was used for quality control analysis of raw genomic sequencing data, fastp(v0.23.2) software was used to remove low-quality data. Through SPAdes_3. 14.1 (https://github.com/ablab/spades) to assemble together, remove < 1000 bp of overlapping fragments; With a RAST (https://rast.nmpdr.org/rast.cgi) statistical information such as salmonella genome size, and the GC content. Whole genome sequencing was performed by Shanghai Berger Medical Technology Co., LTD.

MLST

The results of whole genome sequencing were typed for more site sequence classification through MLST (v2.23.0, https://github.com/tseemann/mlst) , The numbers of each housekeeping gene (aroC, dnaN, hemD, hisD, purE, sucA and thrA) were obtained by alignment with the original allele sequences in the database. and the corresponding sequence type (ST) was obtained by combining them.

Analysis of drug resistance genes and virulence genes

Based on the results of whole genome sequencing analysis, databases CARD (https://card.mcmaster.ca/) and VFDB (http://www.mgc.ac.cn/VFs/) were used to predict the resistance genes and virulence genes using parameters identity>90.0% and coverage>90.0%. Finally, a comparative analysis was conducted on genotype and drug resistance phenotype.

Genetic correlation analysis

Salmonella enterica (Genbank No. NC_003198) were used as the reference genome. KSNP3.01 software was used to construct the core SNPs data set of strains in this study to obtain the single nucleotide polymorphisms (SNPS) of the strains core genome. Sequence alignment and homology analysis were performed on 43 *S. enteritidis* strains using software, and the bootstrap value was set to 1000. Phylogenetic trees were constructed using the maximum likelihood method.

Nucleotide sequence accession numbers

The GenBank accession numbers for the sequences obtained in this study are JBAKMS000000000-JBAKOH000000000 and JBAOJF000000000.

Results

Genome sequencing

The total genome length of 43 strains of *S.enterititis* was 4417739-4757703 bp, and the average GC content was 52.07-52.42%. These lengths and GC contents are consistent with the genomic characteristics of *S. enteritidis*.

MLST molecular typing

MLST analysis based on the whole genome sequence showed that all the 43 *S.enterititis* isolates belonged to ST11 type, and the 7 housekeeping genes were aroC (5), dnaN (2), hemD (3), hisD (7), purE (6), sucA (6) and thrA (11).

Antimicrobial resistance and drug resistance gene analyses

The 43 strains of *S.enteritidis* showed varying degrees of resistance to 17 antibiotics, and resistance rates ranging from 2.33% to 100%, with NAL having the highest resistance rate (100%), followed by AMS (90.70%) and AMS(90.70%).(Table 1). 35 of the 43 *S. enteritidis* strains were resistant to three or more antibacterial drugs, the total multidrug resistance rate was 81.40% (35/43). The 43 *S.enteritidis* strains showed 19 drug resistance spectrum, among which AMP-AMS-NAL-STR-CT and

AMP-AMS-NAL-STR-TET-CT were the predominant drug resistance spectrum, which contained 8 *S.enteritidis* strains, respectively.*S.enteritidis*, which is resistant to 5 antibiotics, accounts for the highest proportion among multidrug-resistant strains, accounting for 31.43% (11/35). The most resistant strain was detected in 2023 (S2023828), which is resistant to all 17 drugs. (Table 2).

Analysis of drug resistance genes indicated that all 43 strains of *Salmonella enteritidis* carried drug resistance genes, and a total of 23 drug resistance genes were predicted. Among them, 14 resistance genes were common to 43 strains of Salmonella enteritidis, including bacA, mdtK, marA, AAC(6')-Iy, cpxA, golS, mdsA, mdsB, mdsC, sdiA, acrB, emrR, H-NS and CRP (FIG 1). The genes with more than 7 antimicrobial resistance were marA, sdiA, acrB, golSmdsA, mdsB, and mdsC, of which marA had the highest carrying rate, (Table 3).

Table 1 Antimicrobial susceptibility testing of 43 strains of *Salmonella enteritidis* 17 antimicrobial agents in 2021-2023

antimicrobial	sensitive（n,%）	intermediate（n,%）	resistant（n,%）
AMP	4(9.30)	0(0.00)	39(90.70)
AMS	4(9.30)	12(27.91)	27(62.80)
CAZ	40(90.2)	0(0.00)	3(6.98)
CZA	41(95.35)	0(0.00)	2(4.65)
CTX	36(83.72)	37(25.34)	7(16.28)
ETP	42(97.67)	0(0.00)	1(2.33)
MEM	42(97.67)	0(0.00)	1(2.33)
NAL	0(0.00)	0(0.00)	43(100)
CIP	42(97.67)	0(0.00)	1(2.33)
AZM	39(90.70)	0(0.00)	4(9.30)
AMI	42(97.67)	0(0.00)	1(2.33)
STR	10(23.26)	6(13.95)	27(62.79)
TET	27(62.80)	0(0.00)	16(37.21)
TIG	42(97.67)	0(0.00)	1(2.33)
CHL	39(90.70)	0(0.00)	4(9.30)
SXT	38(88.37)	0(0.00)	5(11.63)
CT	12(27.91)	0(0.00)	31(72.09)

Table 2 Drug resistant spectrum of 43 strains of *Salmonella enteritidis* to 17 antimicrobial agents in 2021-2023

Antibiotic resistant species (species)	Drug-resistant spectrum	Number of isolate (n)	The percentage (%)
1	NAL	3	6.98
2	AMP-NAL	4	9.30

	NAL-STR	1	2.33
3	AMP-NAL-CT	4	9.30
4	AMP-NAL-AMS-CT	2	4.65
	AMP-AMS-NAL-STR	2	4.65
	AMP-NAL-STR-CT	1	2.33
	AMP-NAL-SXT-CT	1	2.33
5	AMP-AMS-NAL-STR-TET	1	2.33
	AMP-AMS-NAL-STR-CT	8	18.60
	AMP-AMS-NAL-CTX-TET	1	2.33
	AMP-CAZ-CTX-NAL-CT	1	2.33
6	AMP-AMS-NAL-STR-TET-CT	8	18.60
	AMP-AMS-NAL-STR-SXT	1	2.33
	AMP-AMS-CTX-NAL-TET-CT	1	2.33
9	AMP-AMS-CTX-NAL-AZM-STR-TET-CHL-CT	1	2.33
	AMP-AMS-CTX-NAL-AZM-STR-TET-CHL-SXT	1	2.33
12	AMP-AMS-CAZ-CZA-CTX-NAL-AZM-STR-TET-CHL-SXT-CT	1	2.33
17	AMP-AMS-CAZ-CZA-CTX-ETP-MEM-NAL-CIP-AZM-AMI-STR-TET-TIG-CHL-SXT-CT	1	2.33

Table 3 Drug resistance genotyping and drug resistance of *Salmonella enteritidis*

GENE	GENE_COUNT	RESISTANCE_COUNT	RESISTANCE
bacA	43	1	peptide
mdtK	43	1	fluoroquinolone
marA	43	12	carbapenem;cephalosporin;cephamycin;fluoroquinolone;glycylcycline;monobactam;penam;penem;phenicol;rifamycin;tetracycline;triclosan
AAC(6')-Iy	43	1	aminoglycoside
cpxA	43	2	aminocoumarin;aminoglycoside
golS	43	7	carbapenem;cephalosporin;cephamycin;monobactam;penam;penem;phenicol
mdsA	43	7	carbapenem;cephalosporin;cephamycin;monobactam;penam;penem;phenicol
mdsB	43	7	carbapenem;cephalosporin;cephamycin;monobactam;penam;penem;phenicol
mdsC	43	7	carbapenem;cephalosporin;ce

			phamycin;monobactam;penam;penem;phenicol
sdiA	43	8	cephalosporin;fluoroquinolone;glycylcycline;penam;phenicol;rifamycin;tetracycline;triclosan
acrB	43	8	cephalosporin;fluoroquinolone;glycylcycline;penam;phenicol;rifamycin;tetracycline;triclosan
emrR	43	1	fluoroquinolone
H-NS	43	6	cephalosporin;cephamycin;fluoroquinolone;macrolide;penam;tetracycline
CRP	43	3	fluoroquinolone;macrolide;penam
APH(6)-Id	28	1	aminoglycoside
APH(3")-Ib	28	1	aminoglycoside
TEM-1	26	4	cephalosporin;monobactam;penam;penem
sul2	17	1	sulfonamide
tet(A)	11	1	tetracycline
FosA3	2	1	fosfomycin
dfrA17	2	1	diaminopyrimidine
aadA5	2	1	aminoglycoside
CTX-M-14	1	1	cephalosporin

Fig 1. Drug resistance genes detected in the examined 43 *S. enteritidis* isolates in this study.

Virulence genes

Through comparison and analysis of the VFDB database, it was found that 43*S. enteritidis* strains involves 108 virulence genes and involving 20 pathogenic mechanisms. Among them, 96 virulence genomes were shared by 43 strains of Salmonella enteritidis, and type III secretion system is the main pathogenic mechanism of *S. enteritidis*.(图 2）。

Fig 2. Virulence factors detected in the examined 43 *S. enteritidis* isolates in this study.

Genetic correlation analysis

A maximum likelihood tree based on core SNPs was constructed using

NC:003198 as the reference genome. The results showed that 43 strains of *S.enteritis* were divided into 3 clusters. Cluster 1 contains 10 strains, cluster 2 contains 11 strains, and cluster 3 contains 22 strains. Two strains of *S.enteritis* originating from food are located within cluster 3, and the 10 strains from the concentrated outbreaks formed independent branche, which are also within cluster 3 (FIG 3).

FIG 3. Phylogenetic tree based on cg-SNPs of 43 *S. enteritidis* isolates.

Discussion

S. *enteritis* can cause severe invasive infections [26]. The main clinical manifestation is self-limited acute enteritis, and it can also develop into invasive infections outside the intestine, causing systemic diseases [27]. Therefore, it is of great significance to monitor the drug resistance and analyze the genetic characteristics of *S.enteritidis*, which not only provide scientific basis for clinical treatment, but also provide favorable support for tracing the source of infection and establishing a local Salmonella database.

Currently, cephalosporins and quinolones are important antibiotics for the treatment of Escherichia coli infections [28]. However, there have been reports that some strains have developed resistance to these antibiotics [29]. The results of drug sensitivity test in this study showed that *S.enteritidis* in the Huzhou area is highly resistant, with varying degrees of resistance to all 17 kinds of antibiotics. Among all the 17 antibiotics, NAL, AMP, AMS, and STR showed high resistance, which is consistent with the report by Ferrari et al. [30, 31]. This means that NAL, AMP AMS and STR are no longer suitable as empirical drugs for the treatment of Salmonella infection in Huzhou area. In addition, the situation of multiple drug resistance of *S.enteritidis* in Huzhou area is becoming increasingly severe. 35 of the 43 strains of *S.enteritidis* were resistant to three or more antibiotic drugs, with severe resistance to NAL, AMP, and AMS. There are 26 strains resistant to five or more antibiotics, and the highest spectrum of multiple drug resistance is resistant to all 17 antibiotics. The possible reasons for the emergence of multidrug resistance are the abuse of antibiotics and the horizontal transmission of resistance genes. Therefore, in the future, it is necessary to strengthen the scientific and rigorous clinical medication, reduce the occurrence of drug-resistant bacteria, and prevent the spread of drug-resistant strains. Forty-three strains were highly resistant to fluoroquinolones, β-lactams and aminoglycosides, and their genotypes and phenotypes were basically the same. Hyeon et al.[32] reported that some genes are usually not sufficient to cause changes in the resistant phenotype in Salmonella, unless the strain has mutations that can increase their expression[33,34]. Some strains were resistant but no related drug resistance

genes were detected, while some strains were detected related drug resistance genes but did not show drug resistance, which may be caused by unknown genes and other reasons, which need to be further studied.

With the advancement of bioinformatics analysis technology, WGS technology has been widely used in pathogenic bacteria analysis in recent years, which due to its high resolution, good repeatability, and fast and high-throughput characteristics［35］. It is also becoming more and more common to use bioinformatics to predict serotypes based on *Salmonella* whole genome data. In this study, 43 *S. enteritidis* strains isolated in Huzhou from 2021-2023 were sequenced by whole genome sequencing, and compared them with public database strains to construct a phylogenetic tree based on SNPs. The results showed that 43 strains of *S. enteritidis* were divided into 3 clusters. Among them, 10 strains from the outbreaks were clustered in independent branches, In addition, all 3 clusters contained *S. enteritidis* strains from different regions and/or isolation times, indicating that *S. enteritidis* in Huzhou area were in a sporadic state.

MLST is a classical bacterial molecular typing technique widely used to identify relationships among bacterial clones [36].In this study, MLST analysis shows that all the 43 strains of *S. enteritidis* were ST11 type, including 7 housekeeping genes, which indicated a close correlation between sporadic *S. enteritidis* in Huzhou area.
In addition, the detection rate of *S. enteritidis* virulence factors was high, with 96 (88.89%) virulence genomes were shared by 43 strains of *S.enteritidis* in this study. Some studies have found that there had a positive correlation between virulence genes and the pathogenicity of *Salmonella* [37,38]. Among them, the spv gene carried by plasmids of *Salmonella* are commonly present on virulence plasmids of many *Salmonella* species, including the spvRABCDE gene, which mainly mediates cytotoxicity during macrophage differentiation and apoptosis [39]. In this study, 34 strains of *S. enteritidis* carrying spvRBC, indicating that the carrying rate of toxic plasmids of *S. enteritidis* in Huzhou area may be at a relatively high level.

Conclusion

We analyzed the antimicrobial resistance genes and conducted WGS of *S. enteritidis* strains isolated from diarrhea cases in Huzhou. We assessed antibiotic resistance in *S. enteritidis* in that region as well as the genetic characteristics of *S. enteritidis* strains. This information will lay a foundation for the identification and pathogenicity analysis of sporadic and clustered outbreaks of *S. enteritidis* infections in Huzhou.

Declarations

The authors declare that they have no competing interests.

Funding

Huzhou Science and Technology Bureau Project(2023GY08).

Consent for publication

Not applicable.

Availability of data and materials

All relevant data are within the manuscript.

Authors' contributions

DSX and XFW participated in the design of the study. WY and LPC participated in *S. enteritidis* detection. LPC and WY participated in the sequence analysis and phylogenetic analysis. DSX and LPC drafted the manuscript. All authors read and approved the final manuscript.

Acknowledgments

We thank the outpatients, nurses, and clinicians of the participating hospitals for their cooperation with the study.

References

1. Xun Z, Weijie ZH, Minguo G. Surveillance results of foodborne diseases between 2015 and 2018 in Wuxi[J]. Modern Preventive Medicine, 2019,46(14): 2558-2562.
2. Huamin S, Ye J, Jianping H, et al. Active surveillance of foodborne disease in Nantong city, 2015-2016[J]. Modern Preventive Medicine, 2018, 45(7):1323-1327.
3. Ruie H, Suxia Y, Qiuxiang Z, et al. Active etiological sureillance for foodborne diseases in Shanxi Province, 2015-2016[J]. Chinese Journal of Zoonoses, 2019, 35(9): 852-856.
4. van Asten A J, van Dijk J E. Distribution of "classic" virulence factors among Salmonella spp [J]. FEMS Immunol Med Microbiol, 2005, 44(3): 25 1-259.
5. Xia Y, Li H, Shen YQ. Antimicrobial drug resistance in salmonella enteritidis isolated from edible snakes with pneumonia and its pathogenicity in chickens[J]. Front Vet Sci, 2020, 4(7):463.
6. Romeu MJ，Rodrigues D，Azeredo J. Effect of sub-lethal chemical disinfection on the biofilm forming ability，resistance to antibiotics andexpression of virulence

genes of salmonellaenteritidis biofilm-surviving cells[J]. Biofouling, 2020, 36(1):101-112.
7. Li X L， Singh N， Besheares E, et al. Spatial epidemiology of salmonellosis in Florida, 2009-2018[J]. Front Public Health, 2020, 8:603005.
8. Gal-Mor O, Boyle EC, Grassl GA. Same species, diferent diseases: how and why typhoidal and non-typhoidal Salmonella enterica serovars difer[J]. Front Microbiol. 2014;5:391.
9. Cheng RA, Eade CR, Wiedmann M. Embracing diversity: diferences in virulence mechanisms, disease severity, and host adaptations contribute to the success of nontyphoidal Salmonella as a foodborne pathogen[J]. Front Microbiol. 2019;10:1368.
10. Rahman SIA, Dyson ZA, Klemm EJ, et al. Population structure and antimicrobial resistance patterns of Salmonella Typhi isolates in urban Dhaka， Bangladesh from 2004 to 2016[J]. PLoS Neglect Trop D, 2020, 14(2):e8036.
11. Goldrick BA. Foodbome diseases[J]. AM J Nurs, 2003, 103(3):105-106.
12. Yuanbin Y, Qifa S, Peng Y, et al. PFGE subtype and antimicrobial resistance of Salmonella enteritidis isolated in Ningbo, 2003-2014[J]. Disease Surveillance, 2016, 31(12):1007-1011.
13. Mengyingl H, Jian-huil C, Jin-song Y, et al. Use and assessment of PFGE and MLVA molecular typing of Sa lmonella Enteritidis in Fujian Province,China [J]. Journal of Pathogen Biology, 2021, 16(9):1047-1051,1068.
14. Krueger AL, Greene SA, Barzilay EJ, Henao O, Vugia D, Hanna S, et al. Clinical outcomes of nalidixic acid, ceftriaxone, and multidrug-resistant nontyphoidal salmonella infections compared with pansusceptible infections in FoodNet sites, 2006-2008[J]. Foodborne Pathog Dis. 2014;11:335–41.
15. Monte DF, Lincopan N, Berman H, Cerdeira L, Keelara S, Thakur S, et al. Genomic features of high-priority Salmonella enterica serovars circulating in the food production chain, Brazil, 2000-2016[J]. Sci Rep. 2019;9:11058.
16. Zhanhong Y, Yuekang Z, Qiming L, et al. Characteristics of drug resistance and molecular typing of Salmonellas isolated in Zhongshan, Guangdong in 2017 [J]. China' Tropical Medicine, 2019, 19(5):459-462.
17. Yana S, Jianling L, Guangzhong L, et al. Distribution and drug sensitivity analysis of foodborne pathogens in Jinnan District of Tianjin from 2018 to 2020[J]. Chinese Journal of Food Hygiene, 2021,33(5):548-552.
18、Ashton PM, Nair S, Peters TM, Bale JA, Powell DG, Painset A, et al. Identifcation of *Salmonella* for public health surveillance using whole genome sequencing[J]. PeerJ. 2016;4: e1752.
19. Punina NV, Makridakis NM, Remnev MA, Topunov AF. Whole-genome sequencing targets drug-resistant bacterial infections[J]. Hum Genom. 2015;9:19.

20. Yang SM, Baek J, Kim E, Kim HB, Ko S, Kim D, et al. Development of a genoserotyping method for *Salmonella* infantis detection on the basis of pangenome analysis[J]. Microorganisms. 2020;9:67.
21. Premkrishnan BNV, Heinle CE, Uchida A, Purbojati RW, Kushwaha KK, Putra A, et al. The genomic characterisation and comparison of *Bacillus cereus* strains isolated from indoor air[J]. Gut Pathog. 2021;13:6.
22. Neumann B, Rackwitz W, Hunfeld KP, Fuchs S, Werner G, Pfeifer Y. Genome sequences of two clinical *Escherichia coli* isolates harboring the novel colistin-resistance gene variants mcr-1.26 and mcr-1.27[J]. Gut Pathog. 2020;12:40.
23. 23. Wagner E, Zaiser A, Leitner R, Quijada NM, Pracser N, Pietzka A, et al. Virulence characterization and comparative genomics of Listeria monocytogenes sequence type 155 strains[J]. BMC Genom. 2020;21:847.
24. Deshun Xu, Liping Chen, Zhonghao Lu, et al. Prevalence and Serotyping of Salmonella in Retail Food in Huzhou China[J]. Journal of Food Protection, 2024, 87(2):1-5.
25. Foodborne disease surveillance system in Zhejiang province. http://10.44.7.215/DataAuditReport/Sample/AuditQuery
26. Yanmei W, Xiang H, Dongyu Z, et al. Serological typing and molecular typing of 588 foodborne Sal monella isolates from 2017 to 2018 surveillance in Jiangsu Province[J]. Jiangsu J Prev Med, 2021, 32(6):666-668.
27. Guolu S. To analyze the clinical characteristics and risk factors for bacteremia in children with non-typhoidal Salmonella infection[D]. Chongqing: Chongqing Medical University, 2020.
28. Zhan ZQ, Xu XB, Gu Z, et al. Molecular epidemiology and antimicrobial resistance of invasive non-typhoidal Salmonella in China, 2007-2016[J]. Infect Drug Resist, 2019, 12: 2885-2897.
29. Kim SH, Sung GH, Park EH, et al. Serotype distribution and antimicrobial resistance of salmonella isolates in Korea between 2016 and 2017[J]. Ann Lab Med, 2022,42(2):268-273.
30. Ferrari RG, Rosario DKA, Cunha-Neto A, et al. World wide epidemiololgy of Salmonella serovars in animal-based foods :a meta analysis[J]. Appl Env Microbiol, 2019, 85(14): e00591-19.
31. SHI Ai-ping1, NI Jia-min2, SHI Ju-ping1, et al. Molecular typing and drug resistance monitoring of Salmonella enteritidis in patients with diarrhea[J]. Occup and Health, Jun. 2022, 38(12): 1624-1633.
32. Hyeon JY, Li ST, Mann DA, et al. Whole-genome sequencing analysis of Salmonella enterica serolype Enteritidis isolated from poultry sources in South Korea, 2010-2017[J]. Pathogens , 2021 ,10(1): 45. DOI: 10.3390/ pathogensl 00 10045.

33. Salipante SJ, Hall BG. Determining the limits of the evolutionary potential of an antibiotic rssistance gene [J]. Mol Biol Evol, 2003, 20(4): 653-659. DOI: 10. 1093/ molbev/ msg074.
34 Zheng Zhibei, Yu Hua, Chen Qi, et al. Antimicrobial resistance and genomic characteristics of Salmonella enterica serovar Derby isolates from clinical patients and food sources in Hangzhou[J]. Chin J Microbiol Immunol, 2023, 43(2): 115-122.
35. Haijian Z, Biao Z. Progress in application research of bacterium genome-based subtyping[J]. Disease Surveillance, 2016,31(8):668-675.
36. Huang J, Zhao Z, Zhang Q, Zhang SH, Chen M, Qiu H, et al. Phylogenetic analysis reveals distinct evolutionary trajectories of the fluoroquinolones resistant Escherichia coli ST1193 from Fu zhou, China[J]. Front Microbiol, 2021, 12:746995.
37. Rui D. Isolation and identification of Salmonella isolated from eggs and detection of drug resistance genes and virulence genes[D]. Northwest A&F University, 2012.
38. Delin W. Correlation analysis of resistance, pathogenicity, and virulence genes of Salmonella isolated from chicken sources[D].Harbin: Northeast Agricultural University, 2014.
39. Yang J, Rui H. Studies on the plasmid virulence gene spv of Salmonella SPP[J]. Foreign medicine (Branch of Epidemiology and Infectious Diseases), 2004, 31(2):119-120, 124.

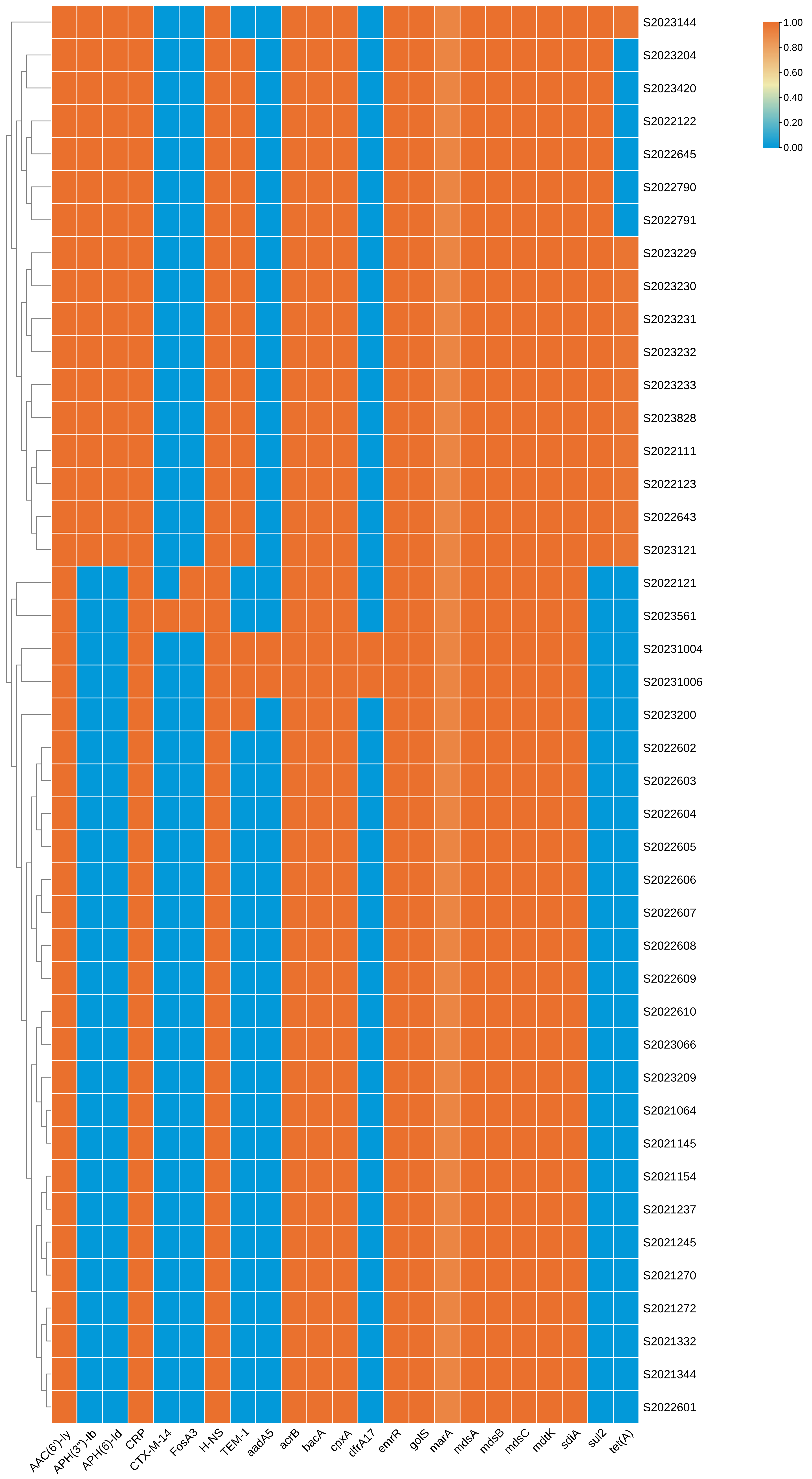
1.00
0.80
0.60
0.40
0.20
0.00
S2023144
S2023204
S2023420
S2022122
S2022645
S2022790
S2022791
S2023229
S2023230
S2023231
S2023232
S2023233
S2023828
S2022111
S2022123
S2022643
S2023121
S2022121
S2023561
S20231004
S20231006
S2023200
S2022602
S2022603
S2022604
S2022605
S2022606
S2022607
S2022608
S2022609
S2022610
S2023066
S2023209
S2021064
S2021145
S2021154
S2021237
S2021245
S2021270
S2021272
S2021332
S2021344
S2022601
AAC(6')-Iy
APH(3'')-Ib
APH(6)-Id
CRP
CTX-M-14
FosA3
H-NS
TEM-1
aadA5
acrB
bacA
cpxA
dfrA17
emrR
golS
marA
mdsA
mdsB
mdsC
mdtK
sdiA
sul2
tet(A)

Click here to access/download;Figure;FEG 2.pdf

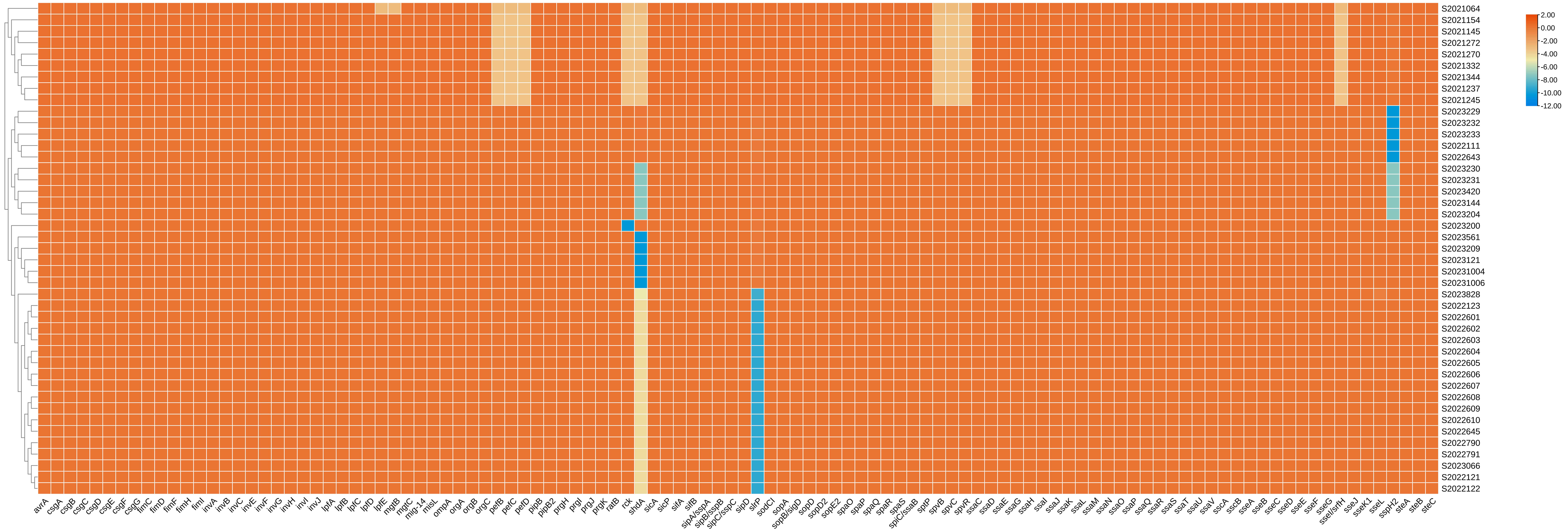

Click here to access/download;Figure;FIG 3.doc

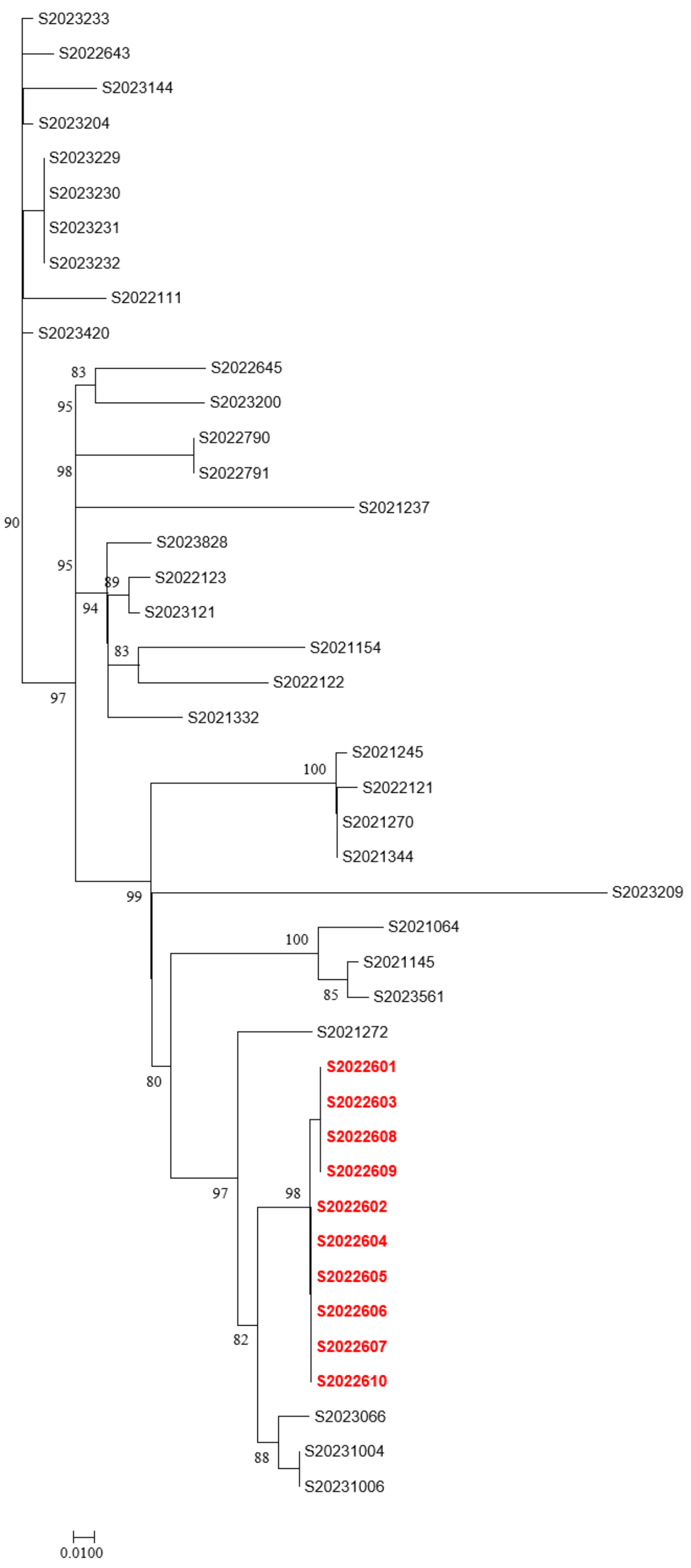

FIG.3 **Phylogenetic tree based on cg-SNPs of 13 *Campylobacter* spp isolates.**

Click here to access/download
Supporting Information
Supplementary Material.docx
