# Supplementary material for: Antimicrobial resistance and genome characteristics of Salmonella enteritidis from Huzhou, China
Source: PLoS One. 2024 Jun 4;19(6):e0304621. doi: 10.1371/journal.pone.0304621 (PMC11149840; doi:10.1371/journal.pone.0304621)
Supplement: Attachment — Submitted filename: PONE-D-24-09010 REVIEWERS COMMENT.pdf [file pone.0304621.s008.pdf]

Antimicrobial resistance and genome characteristics of Salmonella enteritidis from Huzhou, China

REVIEW

Can authors please justify the article?

Kindly proof read and ensure proper spacing after a comma or full stops. Pay attention to details!

INTRODUCTION

Line 41 "Salmonella is a zoonotic pathogen. It is also an important pathogenic factor 42 causing foodborne diseases [2,3] and the main cause of bacterial food poisoning [4]. 43 Contaminated food and water source or infected individuals by Salmonella can 44 become the source of infection and spread to the outside, posing a serious threat to 45 human and animal health[1]".

Any reason for starting the referencing numbers with 2, 3 and 4 before "1"? If none please correct it accordingly.

Line 48 – 49 "gastroenteritis type, septicemia type (typhoid type) and 49 local infection type"

Kindly reference this statement

Authors need to state clearly the justification for the study as dearth of information of the said topic in the region studied.

METHODOLOGY

The authors need to cite references for the methodology "as described by ……" for reproducibility.

Lines 86 – 88 Ethics statement

This study was approved by the human research ethics committee of the Huzhou Center for Disease Control and Prevention.

kindly state the approval number

Line 151 "and the 7 housekeeping genes were aroC 152 (5), dnaN (2), hemD (3), hisD (7), purE (6), sucA (6) and thrA (11)."

These values when added gives 40 as against 43 stated. Any reason for the discrepancy?

Nothing was said about the statistical analysis in the methodology or none was carried out?

RESULT

Can authors confirm the species of Salmonella especially in the result because there are several confusing names?

If authors are not stating a specific species or simple sp. there is no need italicizing the Salmonella

REFERENCES

While writing the authors of articles cited, kindly write out the names of all authors and desist from using et al., in the reference section. Check online for the names of all authors. If truly these articles were downloaded and read before citing them, the authors should have all the names of the authors of articles cited in this study. See lines 286, 289, 301, 310, 314, 317, 320, 324, 327, 330, 333, 338, 342, 348, 351, 356, 362, 365, 368,371, 374, 381, 387

Accept after minor corrections.
