# Supplementary material for: Antimicrobial resistance and genome characteristics of Salmonella enteritidis from Huzhou, China
Source: PLoS One. 2024 Jun 4;19(6):e0304621. doi: 10.1371/journal.pone.0304621 (PMC11149840; doi:10.1371/journal.pone.0304621)
Supplement: Attachment — Submitted filename: Response to Reviewers.doc [file pone.0304621.s010.doc]

Dear  editors: Thank you very much for your letter and the comments from the referees about our paper submitted to PLOS ONE (PONE-D-24-09010).

We have carefully addressed all of the comments from the reviewers, as outlined in the point-by-point responses attached below. We hope that you find the revised manuscript now acceptable for publication in PLOS ONE.

Thanks for your attention

Yours Sincerely

Deshun Xu

Responses to the editor’s comments:

(**Q:** as comments, **A:** as our responses)

**Q:** However, the authors made a strong statement in lines 75 and 76: "There have been no previous reports on drug resistance and genetic characteristics of S enteriditis in the Huzhou area." Unless the authors are 100% sure there is none, I suggest revising this statement.

**A:** We have put "There have been no previous reports on drug resistance and genetic characteristics of *S enteriditis* in the Huzhou area. "changed to “As far as we know, there have been few previous reports on the drug resistance and genetic characteristics of *S.enteritidis* in the Huzhou area”.

**Q:** Based on a few searches, I realized that some co-authors have indeed contributed to previous works that include drug resistance in the Huzhou area. Nevertheless, these studies did not specifically investigate the genetic characteristics of S. enteriditis.
See articles:
1. Prevalence and Serotyping of Salmonella in Retail Food in Huzhou, China (D. Xu et al., 2024)
2. Characterization of Clinical Salmonella entericas Strains in Huzhou, China (D. Xu et al., 2022)

**A:** These two manuscripts are previous research conducted by our research group. One manuscript focuses on the Prevalence and Serotyping of Salmonella in Retail Food in Huzhou, while the other paper examines the Characterization of Clinical Salmonella entericas Strains in Huzhou. However, it is important to note that these studies did not specifically investigate the genetic characteristics of S. enteriditis.

**Q:** B. Furthermore, a review (though it is not a big issue) of the known genetic characteristics of S. enteriditis in other areas of China would be appreciated.
Example articles:
1. The Resistance and Virulence Characteristics of Salmonella Enteritidis Strain Isolated from Patients with Food Poisoning Based on the Whole-Genome Sequencing and Quantitative Proteomic Analysis (B. Xu et al., 2023)
2. Isolation, Identification, Antimicrobial Resistance, Genotyping, and Whole-Genome Sequencing Analysis of Salmonella Enteritidis Isolated from a Food-Poisoning Incident (Hou et al., 2024)

**A:** These two manuscripts have been cited in the article, with References 35 and 42 respectively.
